# Supplementary material for: Identification of the periplasmic DNA receptor for natural transformation of Helicobacter pylori
Source: Nat Commun. 2019 Nov 25;10:5357. doi: 10.1038/s41467-019-13352-6 (PMC6877725; doi:10.1038/s41467-019-13352-6)
Supplement: Supplementary file 1 — Supplementary Information [file 41467_2019_13352_MOESM1_ESM.pdf]

Supplementary information

Identification of the periplasmic DNA receptor for natural transformation of *Helicobacter pylori*

Damke et al.

Supplementary Figure 1

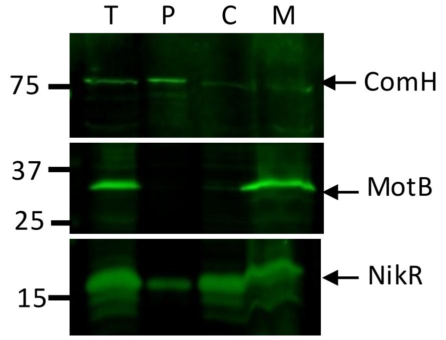

Supplementary Fig. 1. ComH is present in the periplasm. HA-tagged version of ComH was expressed from its own locus and the presence of the protein in the different subcellular compartments was detected by immunoblotting against HA. T: Total extract, P: periplasm, C: cytoplasm, M: membranes.

Supplementary Figure 2

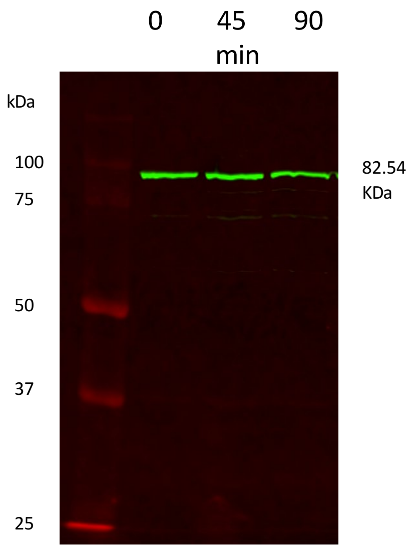

Supplementary Fig. 2. ComH-mCherry expression in *H. pylori.* Bacteria were grown in standard culture conditions used for the co-localisation experiments and at the indicated times harvested. The presence of ComH-mCherry (82.5 kDa) in total cell extracts was verified by Western blot using an antibody against mCherry.

Supplementary Figure 3

a

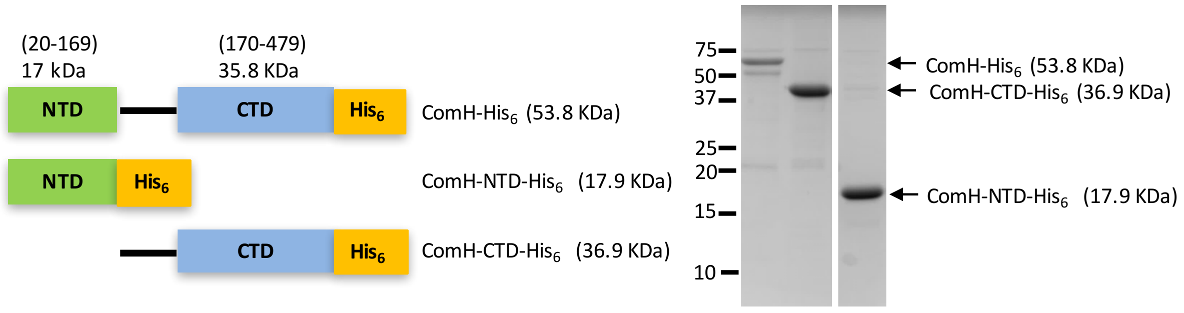

b

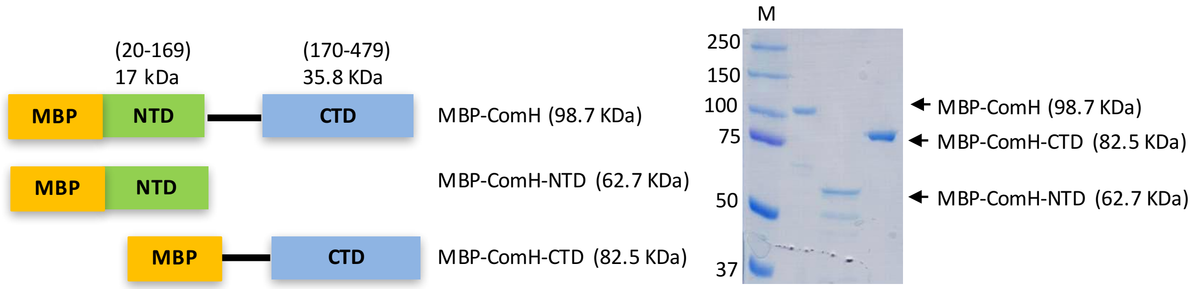

Supplementary Fig. 3. Schematic representation and purification of ComH constructs. The full length ComH protein is composed of 479 amino acids. Based on sequence analysis, the ComH proteins contains an N-terminal signal sequence (amino acids 1-19), N-terminal domain (ComH-NTD) (amino acids 20-169), and C-terminal domain (ComH-CTD) (amino acids 170-479). **a.** C-terminal His_6 fused ComH constructs. The His_6 tagged ComH construct were first purified by Ni-NTA affinity chromatography followed by ion exchange chromatography **b.** N-terminal MBP fused ComH constructs : All the MBP fused ComH constructs were first purified using Amylose affinity chromatography. The full lenght protein was further purified using Resource-S column, MBP-ComH-CTD and MBP-ComH-NTD were further purified using ResourceQ or Heparin column.

Supplementary Figure 4

a

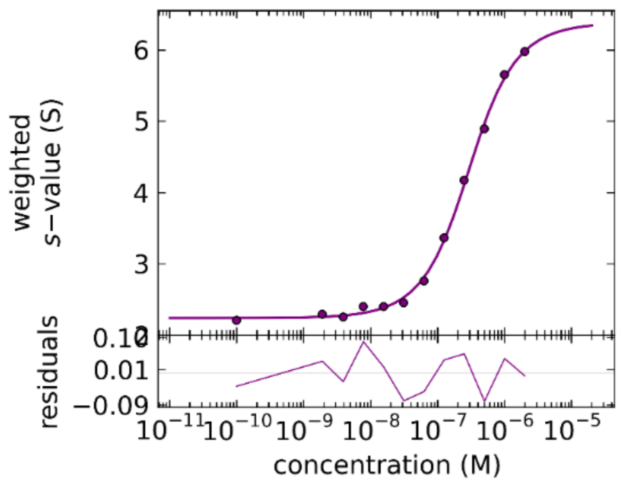

b

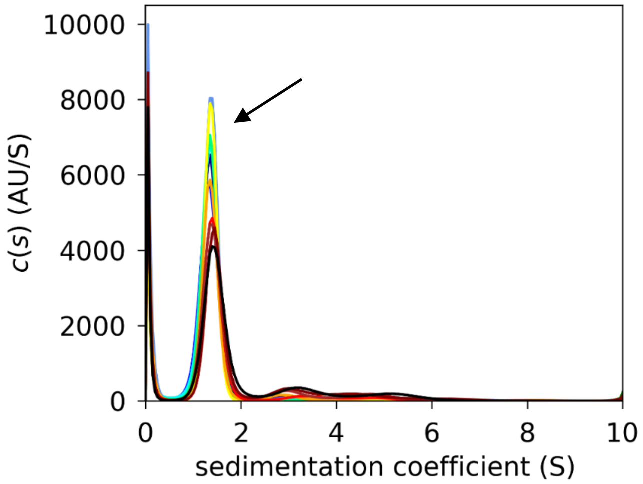

[ComH-His$_6$] (µM) 2, 1.0, 0.5, 0.25, 0.12, 0.06, 0.03, 0.015, 0.006, 0.003

Supplementary Fig. 4. Sedimentation velocity analytical ultracentrifugation (SV-AUC) analysis, analysis of the ComH/DNA interactions. a. isotherm plots, solid circles are the s_w data from the titration series, the full line is the best-fit isotherm with A+B+B⇔AB+B⇔BAB hetero-association model with A corresponding to dsDNA and B corresponding to ComH-His6. b. The interaction between ComH-His6 and a 18nt ssDNA labelled with a FAM in 5' was analysed by isotherm in the same condition than with the dsDNA (Fig 3d). c(s) distributions of 18nt ssDNA was determined in function of ComH-His6 concentration and no difference in the sedimentation profiles is observed which reflects the absence of interaction between the two partners in the condition of AUC experiments.

Supplementary Figure 5

a

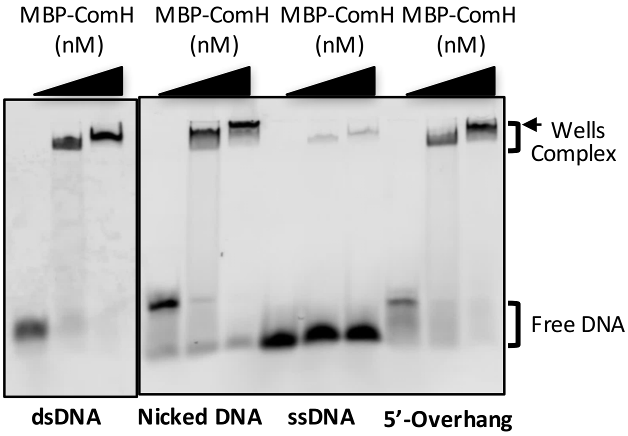

b

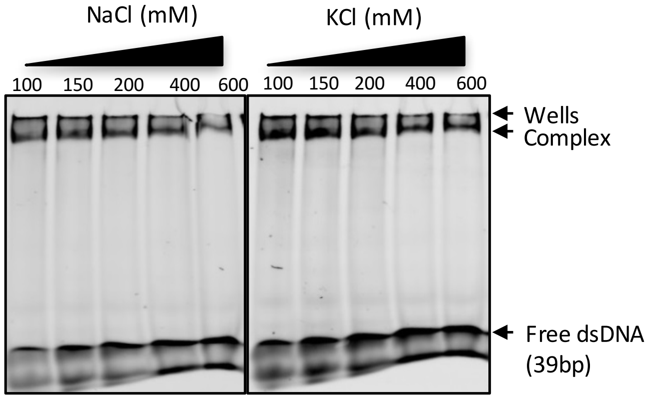

c

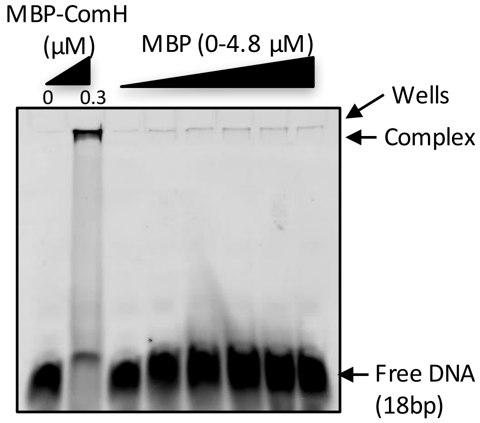

Supplementary Fig. 5. DNA binding characteristics of MBP-ComH. (a) Visualization of nucleoprotein complexes formed by MBP-ComH with indicated DNA substrates (30 nM) . Protein concentrations used were 0, 300, 900 nM. **(b)** Effect of increasing salt concentrations (100-600 mM) on the stability of nucleoprotein complex formed by MBP-ComH (200 nM) with 39bp dsDNA (30 nM). **(c)** EMSA experiment demonstrating no interaction of purified MBP tag alone with tested dsDNA (18bp). All the electrophoretic mobility shift assays were performed by incubating indicated Cy5-labelled DNA substrates with variable concentrations of purified proteins in binding buffer (10 mM Tris-HCl pH 7.5, 50 mM KCl, 0.1 mM Mg, 1 mM DTT, 0.1 µg/µl BSA) for 30 mins on ice. The free DNA substrates and nucleoprotein complexes were resolved by native PAGE (6 %) and the gels were imaged using Typhoon.

Supplementary Figure 6

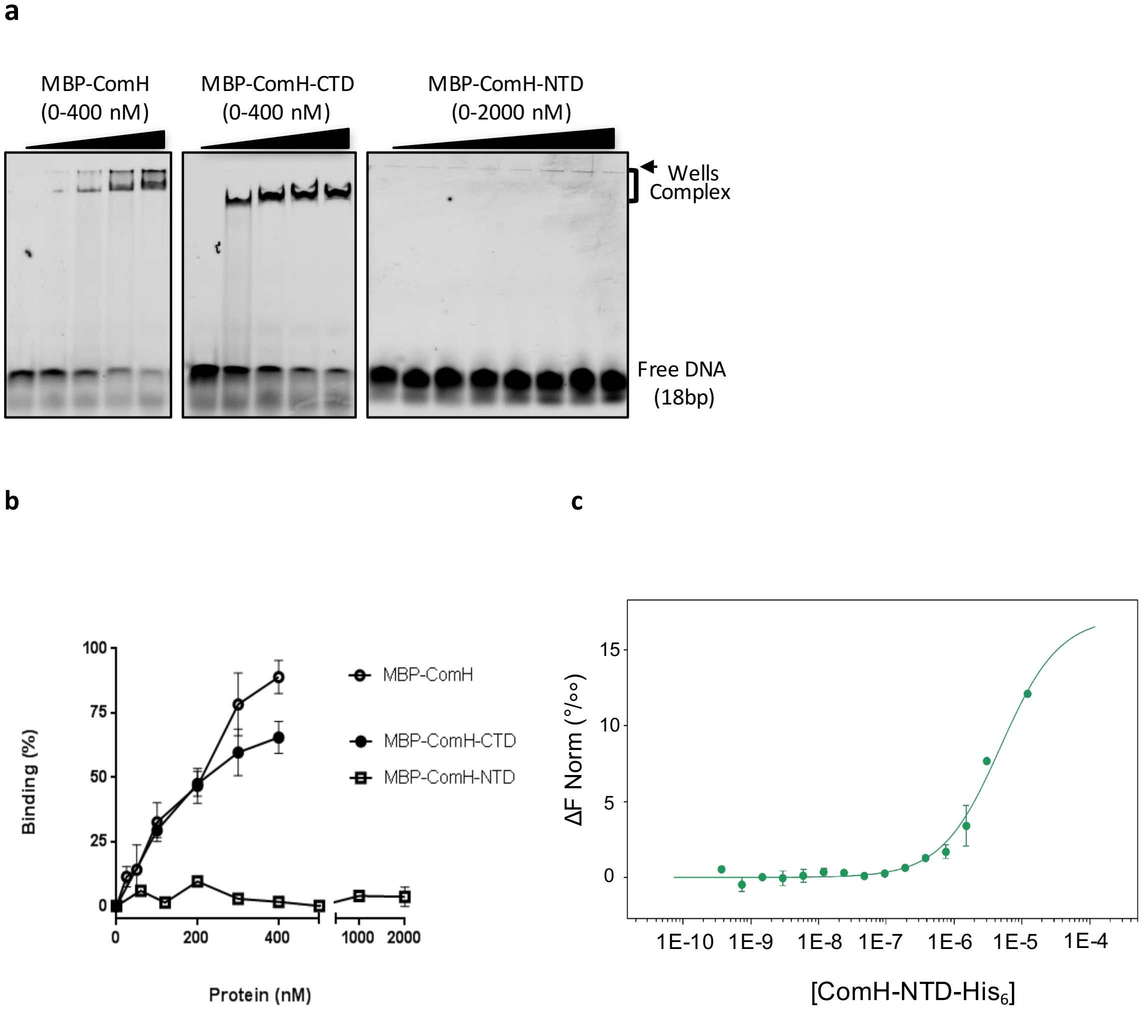

Supplementary Fig. 6. DNA binding affinities of MBP-ComH constructs. (a) Visualization of nucleoprotein complexes formed by MBP-ComH constructs by electrophoretic mobility shift assays. Indicated concentrations of purified proteins were incubated with Cy5-labelled 18bp dsDNA (30 nM) in binding buffer (10 mM Tris-HCl pH 7.5, 50 mM KCl, 0.1 mM Mg, 1 mM DTT, 0.1 µg/µl BSA) for 30 mins on ice. The free DNA substrates and nucleoprotein complexes were resolved by native PAGE (6 %) and the gels were imaged using Typhoon and representative images of resolved gels are shown. Quantification of these gels was performed using ImageJ and the percentage binding was estimated by measuring the depletion in substrate DNA considering DNA without protein as 100 %. The binding percentage as function of protein concentration is shown in **(b)**. Error bars represent standard deviation from two or more different experiments. **(c)** Binding curve of ComH-NTD-His_6 obtained by MST with 18bp DNA with FAM probe in 5'. The K_d is >40µM.

Supplementary Figure 7

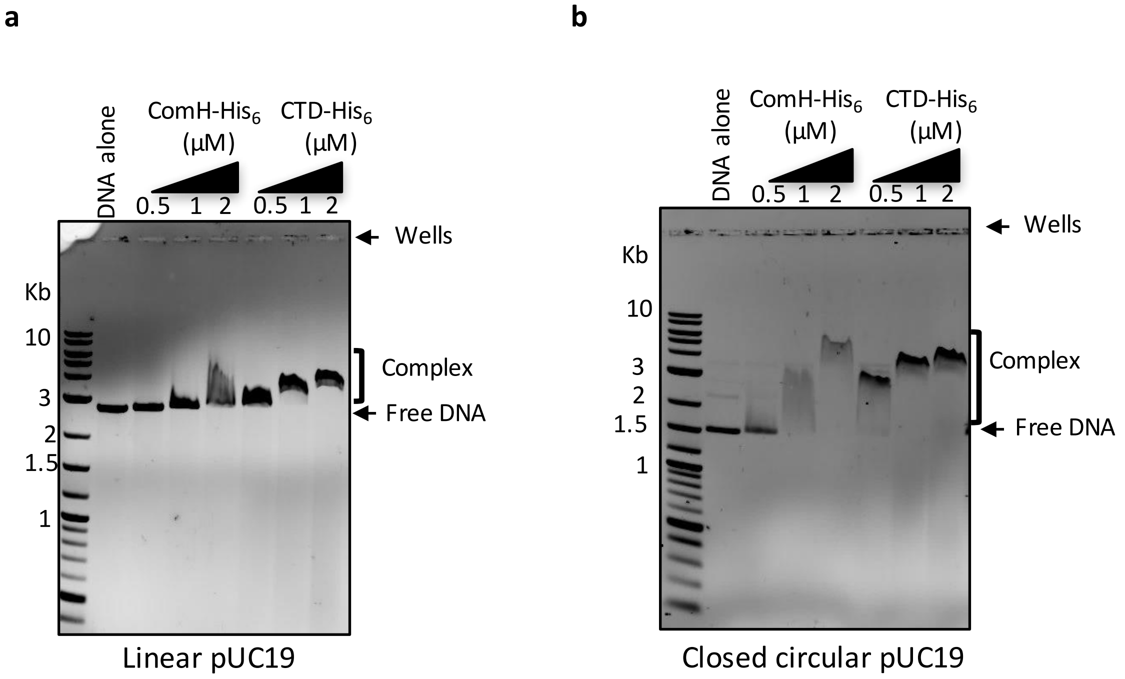

Supplementary Fig. 7. DNA binding by ComH-His$_6$ and ComH-CTD-His$_6$ to (a) linear, and (b) supercoiled pUC19 DNA substrates. Indicated concentrations of purified proteins (0, 0.5, 1, and 2 µM)were incubated with linear and supercoiled pUC19 (7.5 nM) in binding buffer (10 mM Tris-HCl pH 7.5, 50 mM KCl, 0.1 mM Mg, 1 mM DTT, 0.1 µg/µl BSA) for 30 mins on ice. The free DNA substrates and nucleoprotein complexes were resolved by agarose gel electrophoresis (1.2 %) stained with Ethidum bromide (1:10,000).

Supplementary Figure 8

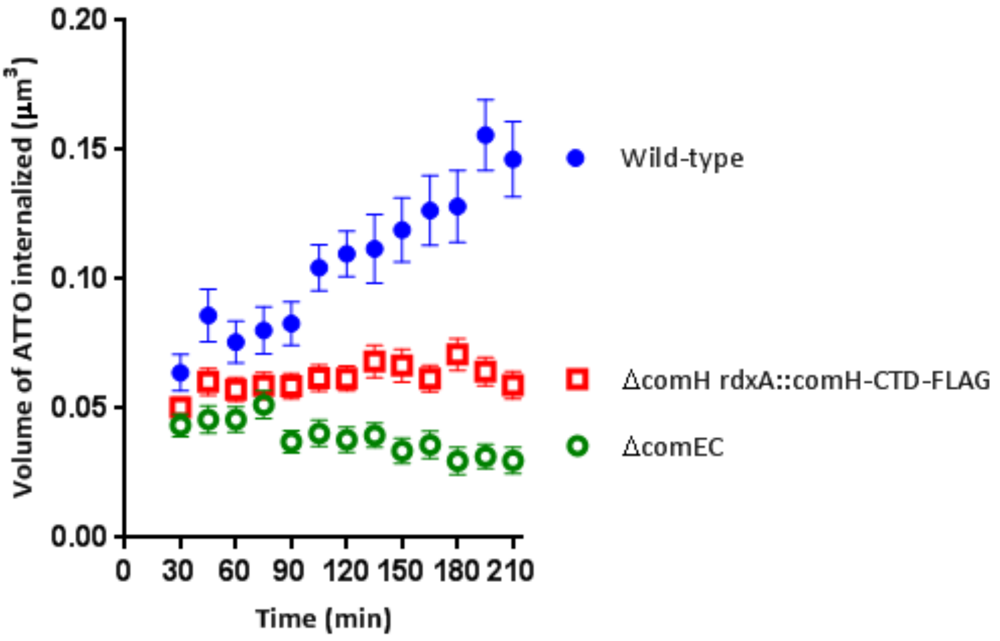

Supplementary Fig. 8. ComH-CTD is not sufficient for translocation of tDNA across the cytoplasmic membrane. tDNA foci (ATTO-550-dUTP labelled 408 bp dsDNA) in GFP expressing bacteria were followed for 3 h by confocal microscopy in live conditions (Supplementary Movies 1-3). The mean ± SEM volumes of DNA internalised were measured by 3D-analysis of individual bacterial cells for wild-type (n=34), *ΔcomEC* (n=39), and *ΔcomH rdxA::comH-CTD-FLAG* (n=66) strains expressing GFP in their cytoplasms. At least two independent experiments were performed for each strain. *P*-values calculated using Kruskal–Wallis statistics indicate that *ΔcomEC* (p<0.0001), and *ΔcomH rdxA::comH-CTD-FLAG* (p=0.0160) curves are significantly different from the wild-type curve.

Supplementary Figure 9

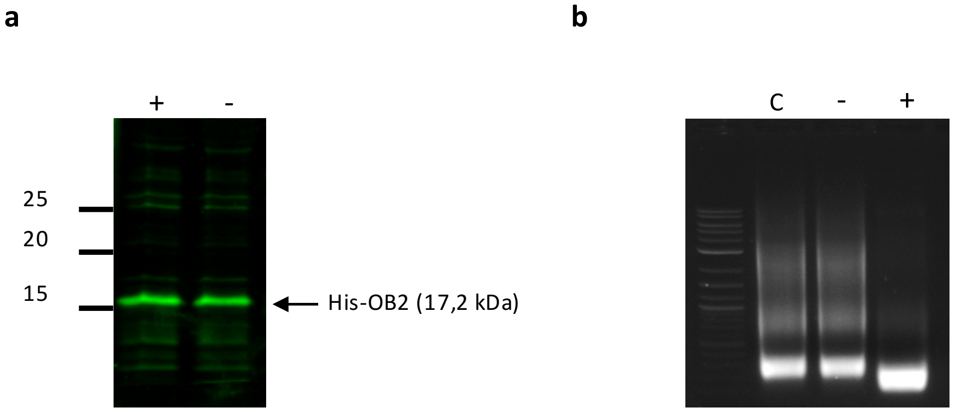

Supplementary Fig. 9. ComH interaction with the ComEC-OB domain does not depend on the presence of DNA. (a) Pulldown experiment as those described for Fig. 6b were carried out with MBP-ComH on samples either treated (+) or not (-) with Benzonase for 30 minutes before putting the samples in contact with the amylose resin. C= DNA before incubation. (b) Agarose gel of the same samples to verify the degradation of DNA from the extracts.

Supplementary Table 1. Natural transformation frequencies determined for *H. pylori* strains. The recombination frequencies of the isogenic streptomycin resistant (StrepR) total genomic DNA were calculated as the number of streptomycin resistance colonies per recipient colony-forming unit. Values correspond to the mean and standard deviation. n: No. of independent determinants. MWU: Mann-Whitney U test.

**Genotype**	**Mean transformation frequencies**	**n**	**Relative value**	**P-value (MWU)**
wild-type	1.60×10^{-3} (1.87×10^{-3})	27	1.00	
comH-BirA-HA	3.51×10^{-5} (4.20×10^{-5})	6	0.022	<0.0001
comH-mCherry	9.59×10^{-4} (7.17×10^{-5})	5	0.6	0.5448

Supplementary Table 2. *H. pylori* strains

**Strain**	**Genotype**	**Source**
LR1	26695	(1)
LR133	26695 *strep*^{R}	(1)
LR293, LR294	26695 *recA::Cm*	(1)
LR827, LR828	26695 *dprA::Cm*	(2)
LR768, LR769	26695 *comB2::Cm*	(3)
LR776, LR777	26695 *comEC::Km*	(3)
LR887	26695 *pUreA-GFPmut2-Km*	(3)
LR901, LR902	26695 *pUreA-GFPmut2-Km comEC::Cm*	(3)
LR957, LR958	26695 *comH::Km*	This work
LR1022, LR1023	26695 *comH::Km rdxA::comH-FLAG-Cm*	This work
LR1116, LR1117	26695 *comH::Km rdxA::comH-CTD-FLAG-Cm*	This work
LR1163, LR1164	26695 *comH::Cm rdxA::comH-CTD-FLAG-Apra pUreA-GFPmut2-Km*	This work
LR1004, LR1005	26695 *comH-mCherry-Cm*	This work
LR1077	26695 *comH-BirA-HA-Cm*	This work

Supplementary Table 3. Primers used in this study

Name	Sequence (5’-3’)	Description
Op81	ATGAAAAAATCCCTTTGTCTGTCTTTC	ComH forward
Op82	TTAAACTTCGTTGAATAAATAATTCCCC	ComH Reverse
Op83	CGGGGTACCGTGCGACTCTTTCAATTCTTTTG AAG	Amplification of p1196
Op84	CGCGGATCCCCAAACAAGACGATCAAGTAGA GC	Amplification of p1196
KpnI-Km-for	GGTACCCGGGTGACTAACTAGGAGG	Amplification of Km resistance cassette
BamH-Km-rev	TCTAGAGGATCCCCGGGTCATTATTC	Amplification of Km resistance cassette
KpnI-Cm-for	CGGGGTACCGCCATATTGTG	Amplification of Cm resistance cassette
BamHI-Cm-rev	CGGGATCCTTACGCCCCGCCC	Amplification of Cm resistance cassette
Op99	CACTTCTTTTCAAATCCCACAACC	Prom-ComH forward
Op100	TTACTTATCGTCGTCATCCTTGTAATCAACTTC GTTGAATAAATAATTCCCC	ComH-FLAG-STOP
Op101	GTTGTGGGATTTGAAAAGAAGTGGTGTGGTA ACAACTCGCTGG	Amplification of p1081
Op102	AGGATGACGACGATAAGTAAGGTACCGCCAT ATTGTGTTGAAAC	Amplification of p1081
Op200	GGCTTGAAGAGGGTTAGAGAAAGTCAG	ComH Leader rev
Op202	CAGCCTTTAGCAATTCCTAGCAAAATAAGTC	ComH Cter 158-479
Op86	GTGTTTTGTTCTTGCTGCATTTTAG	Primers flanking *hp1527*
Op104	GAAACCAGCGAATTGGCTTATTC	ComH internal primer
Op107	GAGCTCGGAGCACCTGCCGC	Linker forward
Op108	TTACGCCCCGCCCTGCCACTC	Cm-reverse
Op105	GCGGCAGGTGCTCCGAGCTCAACTTCGTTGA ATAAATAATTC	ComH-linker reverse
Op106	AGTGGCAGGGCGGGGCGTAAGATGGTGGCT TGAGGCGGAATC	Cm-ComH
Op284	GGACACCCGTTCGCGATTG	Amplification of p1391
Op285	CGTCAGGATGGCCTTCTGC	Amplification of p1391
Op31	ATGGAATTGAATCAACCACCACTC	Amplification of *hp0247*
Op32	TTAACGGCGTTTGGGTTTTTTAG	Amplification of *hp0247*
1197-5’	GTGCCTACTATCAATCAGCTGATTAGAAAAG	Amplification of *hp1197*
1197-3’	TTATTTTTTCTTGTTGTCGGTTGCTTTCTTGTC	Amplification of *hp1197*
OSF342	GAAGCGAGAAAACCCAAGCC	Amplification of *hp1197*

OSF343	CTAGCACGCGCATGGATCGTTCC	Amplification of *hp1197*
Op220	AAGAAGGAGATATACATATGCTTGTGATCGAGCTTTTAGAAGAAATC	pnEA-vH-ComH-forward
Op221	CCGTGGTGGTGGTGGTGGTGAACTTCGTTGAATAAATAATTCCCC	pnEA-vH-ComH-20-479-reverse
Op219	CACCACCACCACCACCACGG	Amplification of pnEA-vH forward
Op226	CATATGTATATCTCCTTCTTAAAGTTAAACAAAATTATTACTAGTGGGG	Amplification of pnEA-vH reverse
Op239	TCCTAGCAAAATAAGTCCCACTAACCACCACCACCACCACCACGGTACCTAATAGTCTAGAGCTAGCCCTAGGAGATCCGGC	Amplification of p1364
Op240	GCCGGATCTCCTAGGGCTAGCTCTAGACTATTAGGTACCGTGGTGGTGGTGGTGGTGGTTAGTGGGACTTATTTTGCTAGGA	Amplification of p1364
Op243	GTTTAACTTTAAGAAGGAGATATACATATGAACGAAACTCAAACGCCAACAAACG	Amplification of p1364
Op244	CGTTTGTTGGCGTTTGAGTTTCGTTCATATGTATATCTCCTTCTTAAAGTTAAAC	Amplification of p1364
Op127	CTTGTGATCGAGCTTTTAGAAGAAATC	ComH-delta leader forward
Op128	CTTCTAAAAGCTCGATCACAAGGAATTCTGAAATGCCCTGAAAATAC	Amplification of pMAL-p2X
Op129	GAATTATTTATTCAACGAAGTTTAACTCTAGAGTCGACCTGCAGG	Amplification of pMAL-p2X
Op198	AGTGGGACTTATTTTGCTAGGAATTGC	Amplification of p1249
Op205	TAATAACTCTAGAGTCGACCTGCAGGCAAG	Amplification of p1249
Op206	GCCCTGAAAATACAGGTTTTC	Amplification of p1249
Op201	AACGAAACTCAAACGCCAACAAACG	Amplification of p1249
HpComEC-30_F	GAAAACCTTTACTTCCAGGGCACAAGTTTGAGCGCTCAAATC	Amplification of HpComEC (30-156)
HpComEC-156_R2	GGGCTAGCTCTAGACTATTAGGATCCTCATAAGTGGTTGATCCCTAGCG	Amplification of HpComEC (30-156)

Supplementary Table 4. Plasmids used in this study

**Name**	**Description**	**Source**
p1196	pJet1.2-*comH*	This study
p1198	pJet1.2-*comH::Km*	This study
p1295	pJet1.2-*comH::Cm*	This study
p1081	pJet1.2-*rdxA::Prom-recA-mCherry-Cm*	Lab. collection
P1235	pJet1.2-*Prom-comH-FLAG-Cm*	This study
p1337	pJet1.2-*Prom-comH-CTD (158-479)-FLAG-Cm*	This study
p1457	pJet1.2-*Prom-comH-CTD (158-479)-FLAG-Apra*	This study
p1225	pJet1.2-end of *comH gene (+- 300 bp)*	This study
p1146	pJet1.2-*nucT-linker-mcherry-Cm (Y82C, S141N)*	Lab. collection
p1236	pJet1.2-*comH-linker-mcherry-Cm*	This study
p1320	pJet1.2-*comH-linker-BirA-HA-Cm*	This study
p1391	pBAD33-*Dronpa-MTSBs*	Lab. collection
p1364	pnEA-vH-*comH (20-479)-His6*	This study
p1384	pnEA-vH-*comH-NTD (20-169)-*His_6	This study
p1381	pnEA-vH-*ComH-CTD (170-479)-*His_6	This study
p1249	pMAL-*p2X-MBP-comH (20-479)*	This study
p1342	pMAL-p2X-*MBP-comH-NTD (20-169)*	This study
p1353	pMAL-p2X *MBP-comH-CTD (170-479)*	This study
p1397	pnEA-vH-*comEC (30-156)-OB-*His_6	This study

Supplementary Table 5. DNA substrates used in this study

**Sequence of oligonucleotides used as DNA substrates**		
**Name**	**Sequence (5'-3')**	**Description (size)**
Op245	AATCACCTGCATTAGTGGAATGCCCTCAAAGGAGAGGGGTTTGTACTAGGGTTTATACGACTACCCCTAGAAAGCCTAACTCGGCTTTAAGAAAGGTTGCCAAAGTTCGTTTGACCAGTAAATTTGAAGTGATCAGTTA	Streptomycin resistant (139-mer) ssDNA for electroporation
Cy5-PNK1	TAGCATCGATCAGTCCTC	5'-Cy5 labelled ssDNA (18-mer)
cPNK1	GAGGACTGATCGATGCTA	(18-mer)
PNK2-TAMRA	GAGGTCTAGCATCGTTAGTCA	5'- TAMRA labelled ssDNA (21-mer)
PNK3	TGACTAACGATGCTAGACCTCGAGGACTGATCGATGCTA	(39-mer)
Cy5-PNK3	TGACTAACGATGCTAGACCTCGAGGACTGATCGATGCTA	5'-Cy5 labelled ssDNA (39-mer)
cPNK3	TAGCATCGATCAGTCCTCGAGGTCTAGCATCGTTAGTCA	(39-mer)
Op249	GTAGTGCTGTAGGAGAATATACGGGCTGCTCGTGTTGACAAGTACTGATG	for SPR, 5'-biotinylated (50-mer)
Op250	CATCACGACATCCTCTTATATGCCCGACGAGCACAACTGTTCATGACTAC	for SPR (50-mer)
**Combination of oligonucleotides used to prepare duplex DNA substrates**		
**DNA substrate**	**Composition**	**Description**
18 bp dsDNA	Cy5-PNK1 + cPNK1	for EMSA
5'-overhang	Cy5-PNK1 + PNK3	for EMSA
Nicked DNA	Cy5-PNK1 + PNK2-TAMRA + PNK3	for EMSA
39 bp dsDNA	Cy5-PNK3 + cPNK3	for EMSA
50 bp dsDNA	Op249 + Op250	for SPR

References

1. Marsin S, Mathieu A, Kortulewski T, Guérois R, Radicella JP. Unveiling novel RecO distant orthologues involved in homologous recombination. PLoS Genet. 2008;4(8).
2. Orillard E, Radicella JP, Marsin S. Biochemical and cellular characterization of Helicobacter pylori RecA, a protein with high-level constitutive expression. J Bacteriol. 2011;193(23):6490–7.
3. Corbinais C, Mathieu A, Kortulewski T, Radicella JP, Marsin S. Following transforming DNA in Helicobacter pylori from uptake to expression. Mol Microbiol. 2016;101(6):1039–53.
