# Supplementary material for: Identification of the periplasmic DNA receptor for natural transformation of Helicobacter pylori
Source: Nat Commun. 2019 Nov 25;10:5357. doi: 10.1038/s41467-019-13352-6 (PMC6877725; doi:10.1038/s41467-019-13352-6)
Supplement: Supplementary file 3 — Description of Additional Supplementary Files [file 41467_2019_13352_MOESM3_ESM.pdf]

Description of Additional Supplementary Files

File Name: Supplementary Movie 1
Description: **Internalisation of fluorescently labelled DNA into the bacterial cytoplasm of a *wild-type* strain.** The internalisation of the ATTO488-labelled DNA into the cytoplasm of GFP expressing *wild-type* cells was followed by live microscopy.

File Name: Supplementary Movie 2
Description: **Internalisation of fluorescently labelled DNA into the bacterial cytoplasm of a *ΔcomEC* strain.** The internalisation of the ATTO488-labelled DNA into the cytoplasm of GFP expressing *ΔcomEC* cells was followed by live microscopy.

File Name: Supplementary Movie 3
Description: **Internalisation of fluorescently labelled DNA into the bacterial cytoplasm of a *ΔcomH* strain expressing ComH-CTD.** The internalisation of the ATTO488-labelled DNA into the cytoplasm of GFP expressing *ΔcomH rdxA::ComH-CTD-FLAG* cells was followed by live microscopy.
